# Supplementary material for: Selective Inhibition of Liver Cancer Cells Using Venom Peptide
Source: Mar Drugs. 2019 Oct 17;17(10):587. doi: 10.3390/md17100587 (PMC6835663; doi:10.3390/md17100587)
Supplement: Supplementary file 1 [file marinedrugs-17-00587-s001.pdf]

Supporting information

Selective inhibition of liver cancer cells using venom peptide

Prachi Anand[1,2,3,4], Petr Filipenko[1], Jeannette Huaman[1,5,6], Michael Lyudmer[1], Marouf Hossain[1], Carolina Santamaria[1], Kelly Huang[1], Olorunseun O. Ogunwobi[1,5,6], Mandë Holford* [1,2,3,4]

[1] Department of Chemistry and Biochemistry, -Hunter College, Belfer Research Building 413 East 69th Street, New York, NY 10021

[2] American Museum of Natural History, Central Park West & 79th St, New York, NY 10024

[3] CUNY Graduate Center Chemistry, Biology, Biochemistry Programs, 365 5th Ave, New York, NY 10016

[4] Weill Cornell Medical College (Biochemistry Department), 1300 York Avenue, New York, NY 10065

[5] Joan and Sanford I. Weill Department of Medicine, Weill Cornell Medical College, 1300 York Avenue, New York, NY 10065;

[6] Department of Biological Sciences, Hunter College, 695 Park Avenue, New York, NY 10065

* Correspondence: mholford@hunter.cuny.edu; Tel.: +1-212-896-0449

Supplementary figures S1- S16 and supplementary table S1.

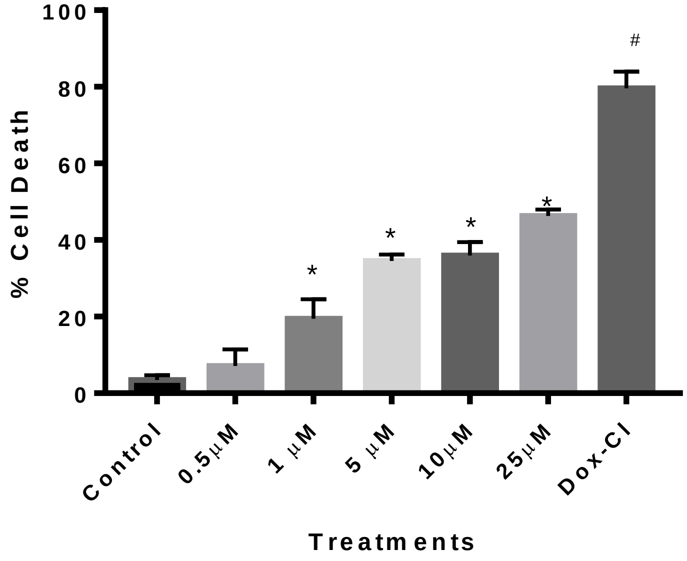

Figure S1. Cytotoxicity of Tv1 on human liver cancer cell line HepG2 Cytotoxicity of Tv1 in HepG2 cells after 48 h treatment of Tv1 showed a significant cell death of 40%. (n=5). The analysis was done using one-way ANOVA and Dunnetts's multiple comparison post-test. *p<0.01, #p<0.001

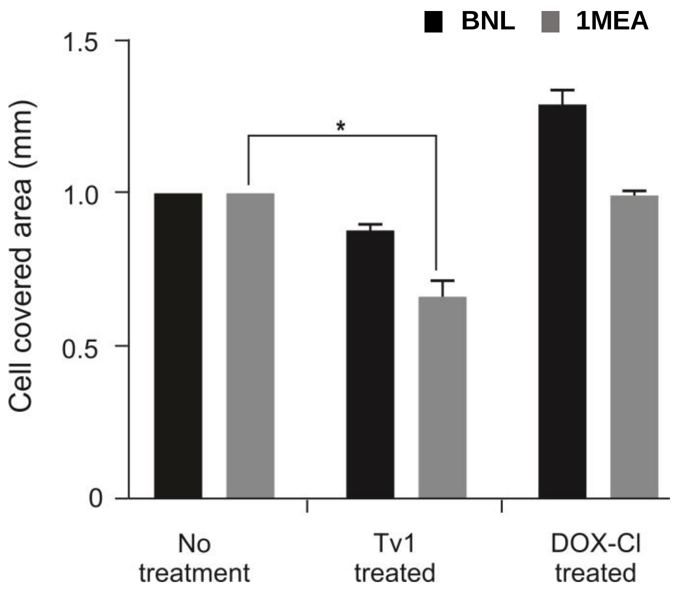

Figure S2. Effect of Tv1 on migration using wound healing assay. BNL (black) and 1MEA (grey) cells that were treated for 6 h with Tv1 (0.5µM) or DOX (0.5 µM). Quantitative analysis of migration assays (% of cell-covered area) revealed that 1MEA cells treated with Tv1 significantly inhibited the growth of cells in the wound. Results expressed as mean ± SEM. *p<0.01, n = 3.

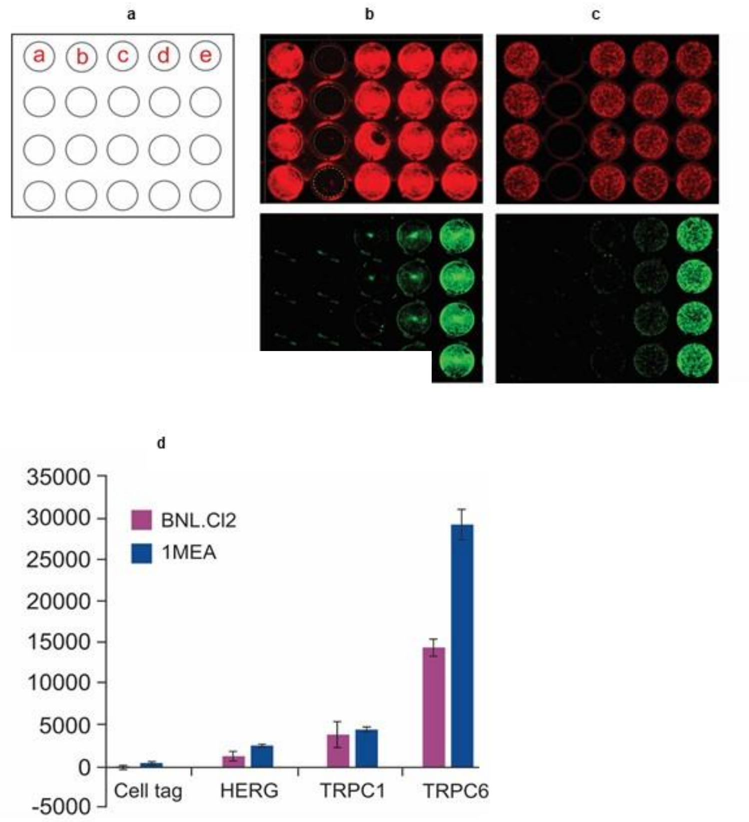

Figure S3. In-cell western analysis for the screening of ion channels in BNL.CL2 and 1MEA cells. (a) Experimental plan for the wells treated with different antibodies. (b) BNL cells (c) 1 MEA cells. (d) Intensities are compared at 800nm channels after normalizing at 700nm channel for cell tag. Cells incubated with TRPC6 antibodies only showed significant presence of channels.

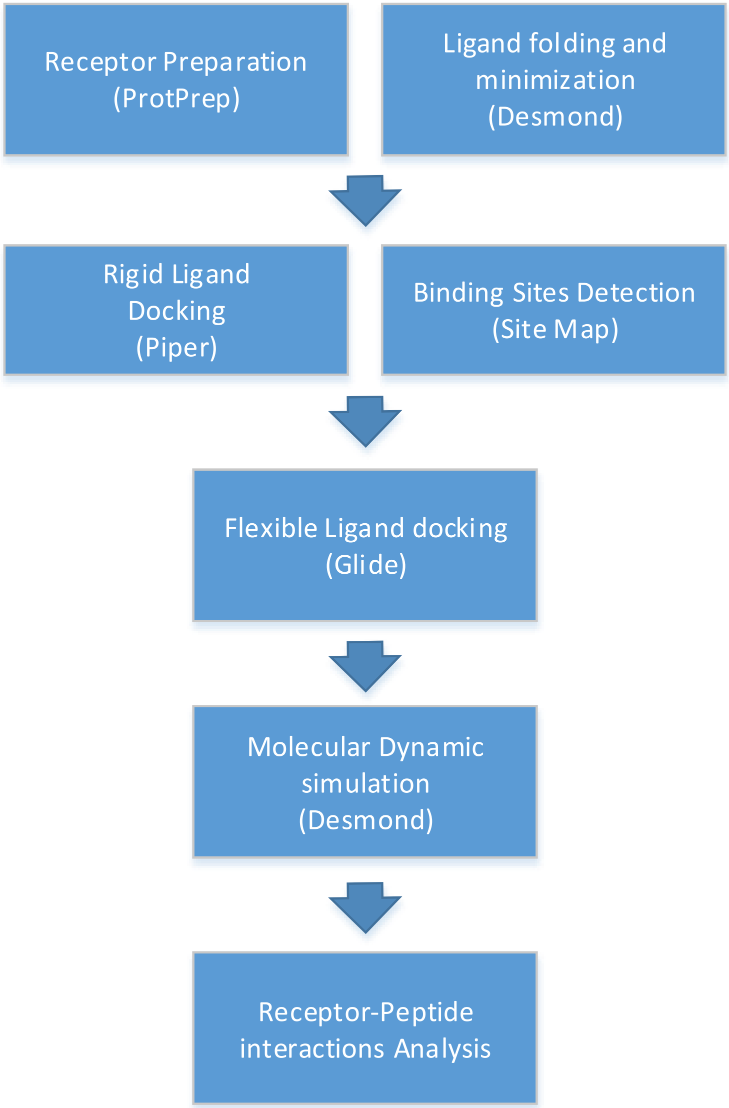

Figure S4. Receptor-ligand preparation pipeline diagram

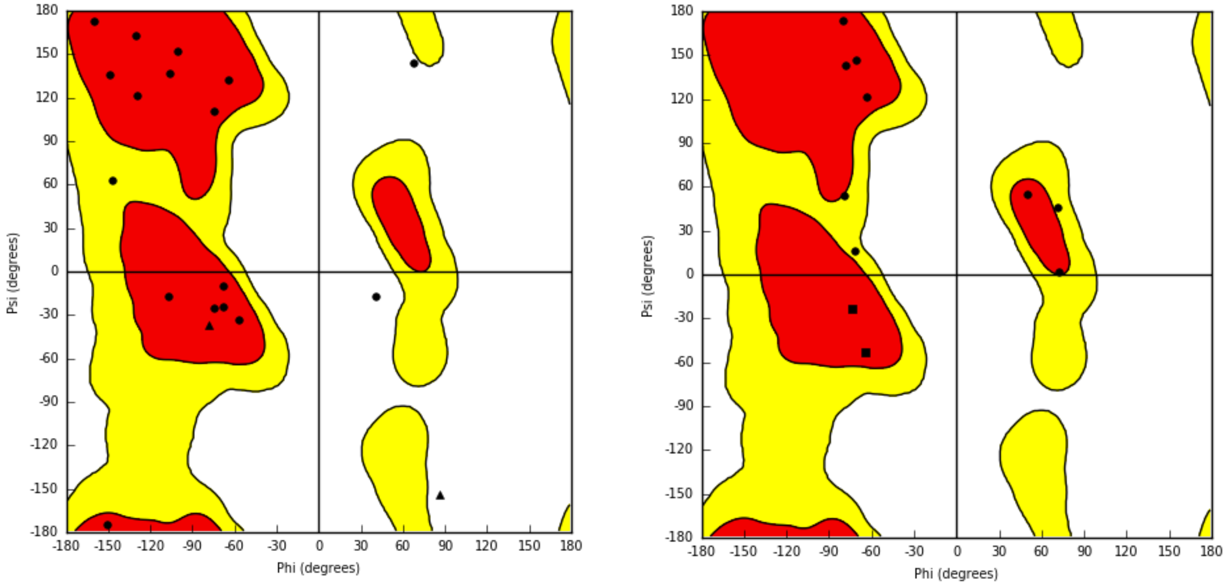

a b

Figure S5. Ramachandran plots of ligand models after protein preparation protocol and 300ns molecular dynamic run. (a) Tv1 (PDB: 2MIX); **(b)** TruncSorC

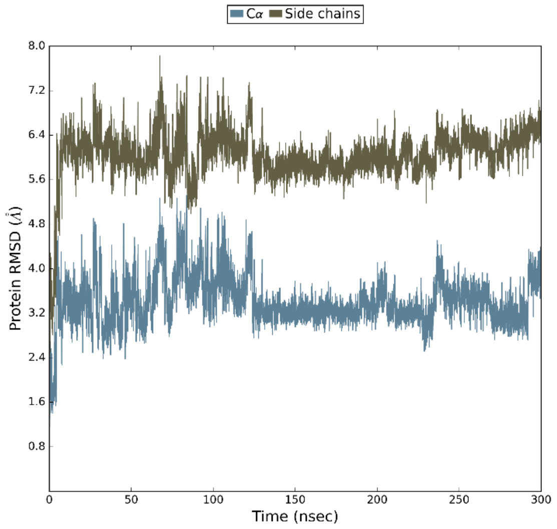

a

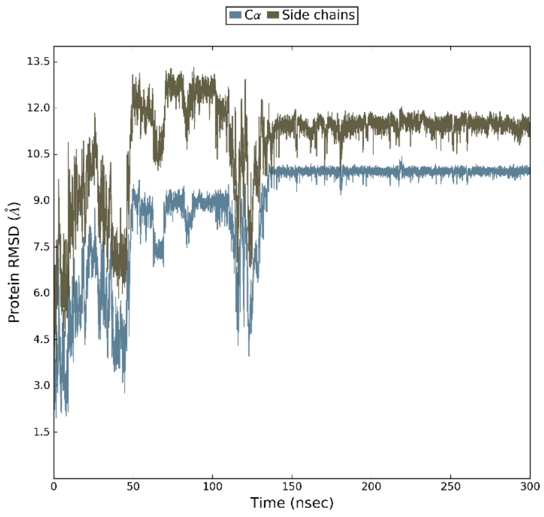

b

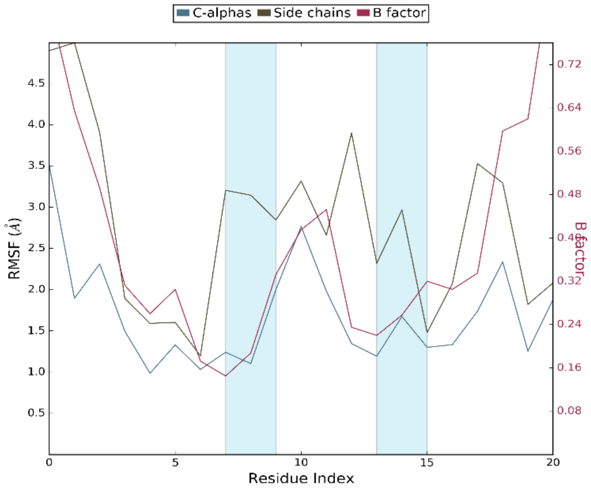

c

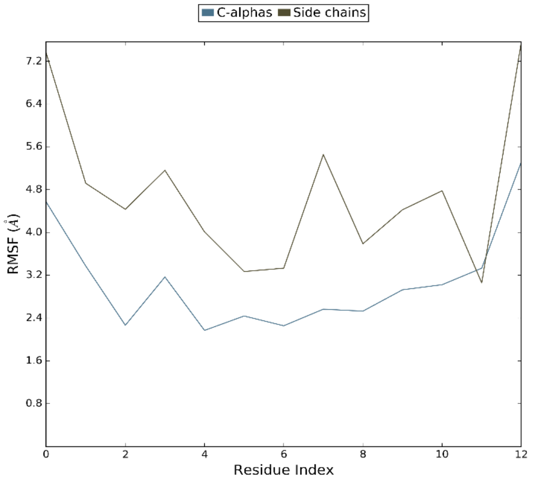

d

Figure S6. Structural analysis of ligands during 300ns molecular dynamic run. (a) RMSD plot of Tv1. (b) RMSD plot of truncSorC. (c) RMSF plot of Tv1 model with residue positions forming a secondary structure marked in light blue. (d) RMSF plot of truncSorC.

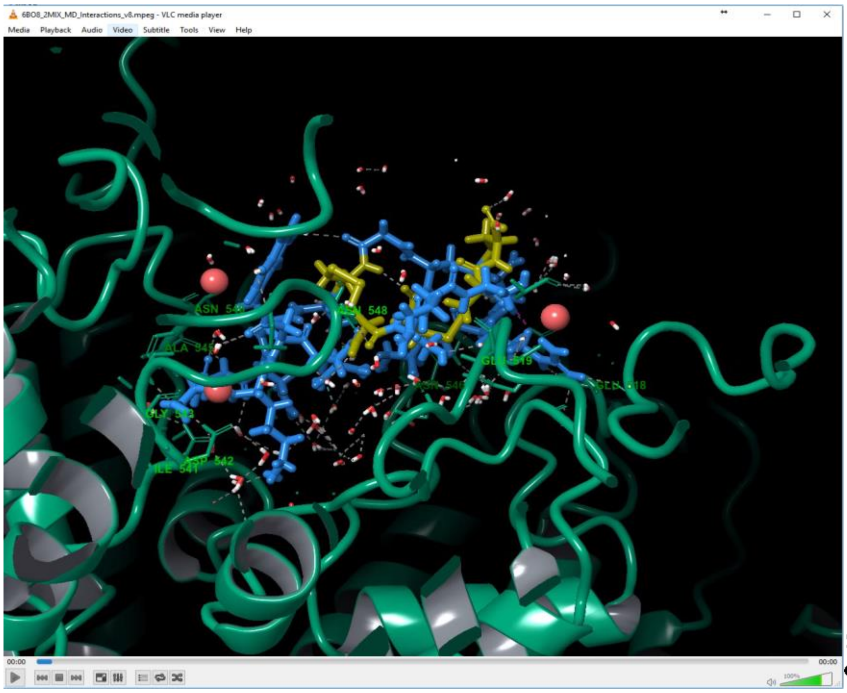

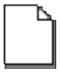

Figure S7. Video recording of receptor-ligand-solvent interactions during 150ns molecular dynamic simulation. Water and salt ions are displayed within 4Å radius from ligand. (Video can be viewed by clicking on the icon)

S8-Tv1-hTRPV6
model in membrane.r

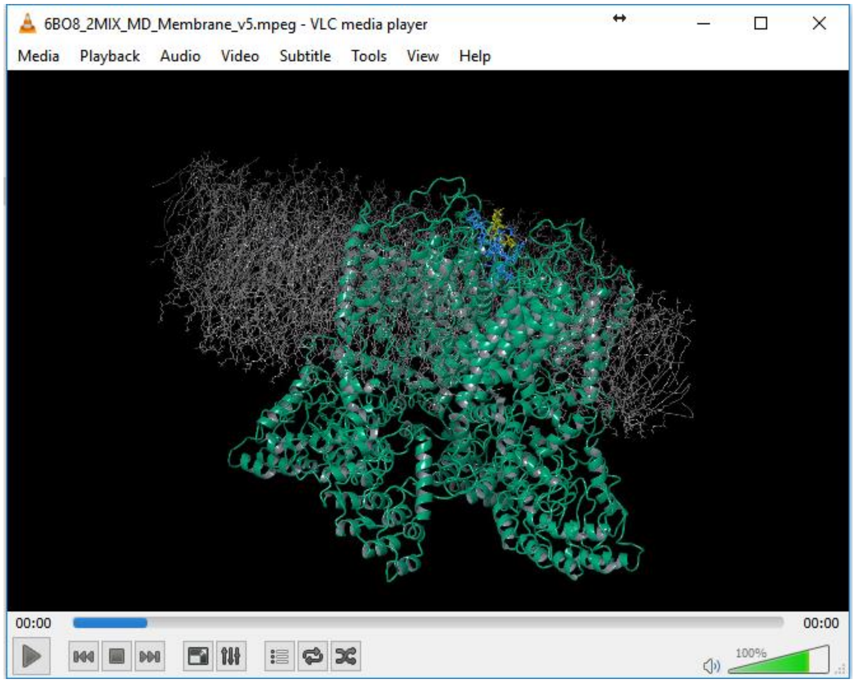

Figure S8. Video recording of Tv1/hTRPV6 model in membrane during 150ns molecular dynamic simulation. Water and salt ions are not displayed. (Video can be viewed by clicking on the icon)

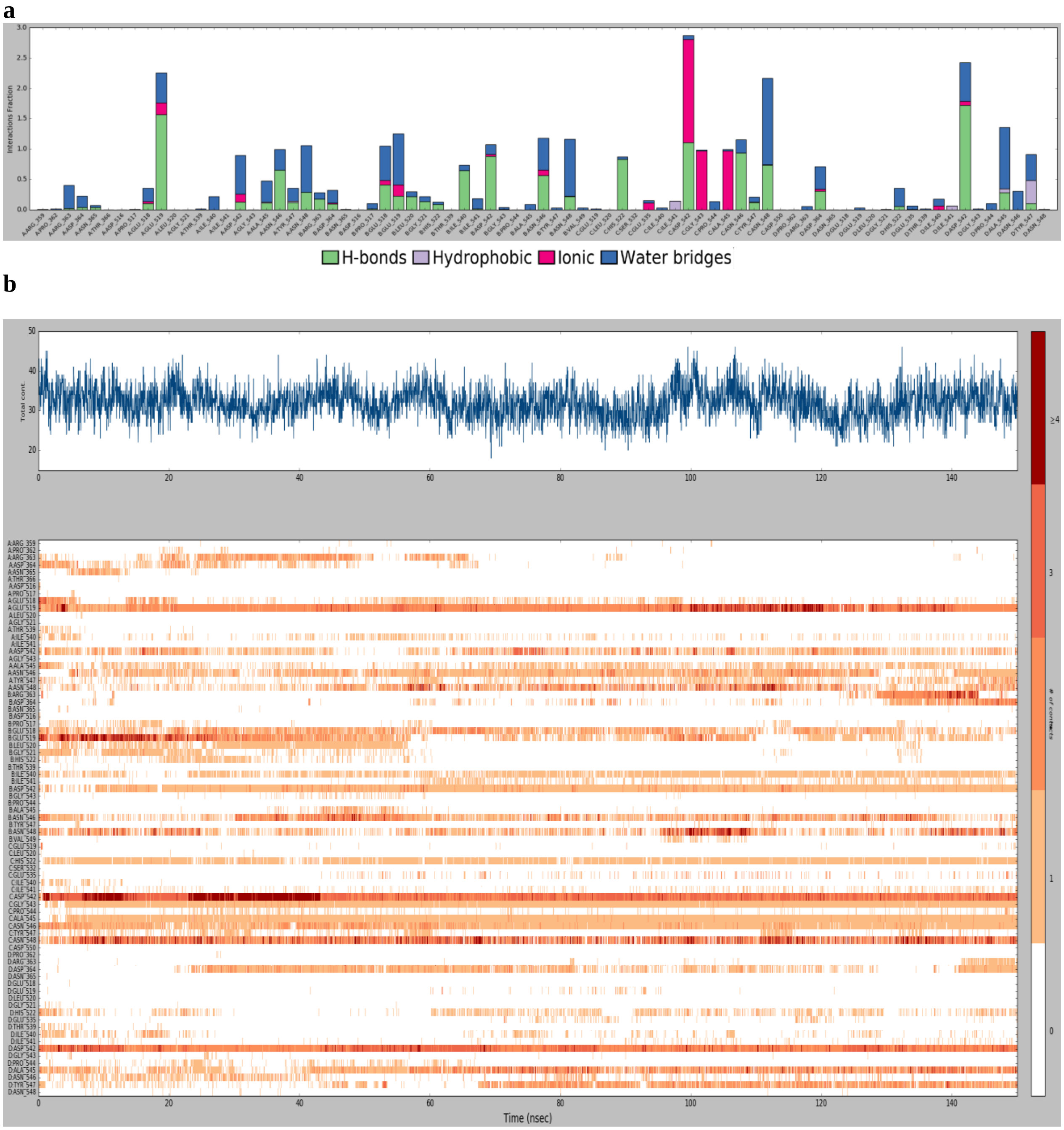

Figure S9. Summary of receptor-ligand interactions for Tv1-hTRPV6 molecular dynamics simulation. **(a)** Normalized over trajectory. **(b)** Timeline representation of all contacts per receptor residue.

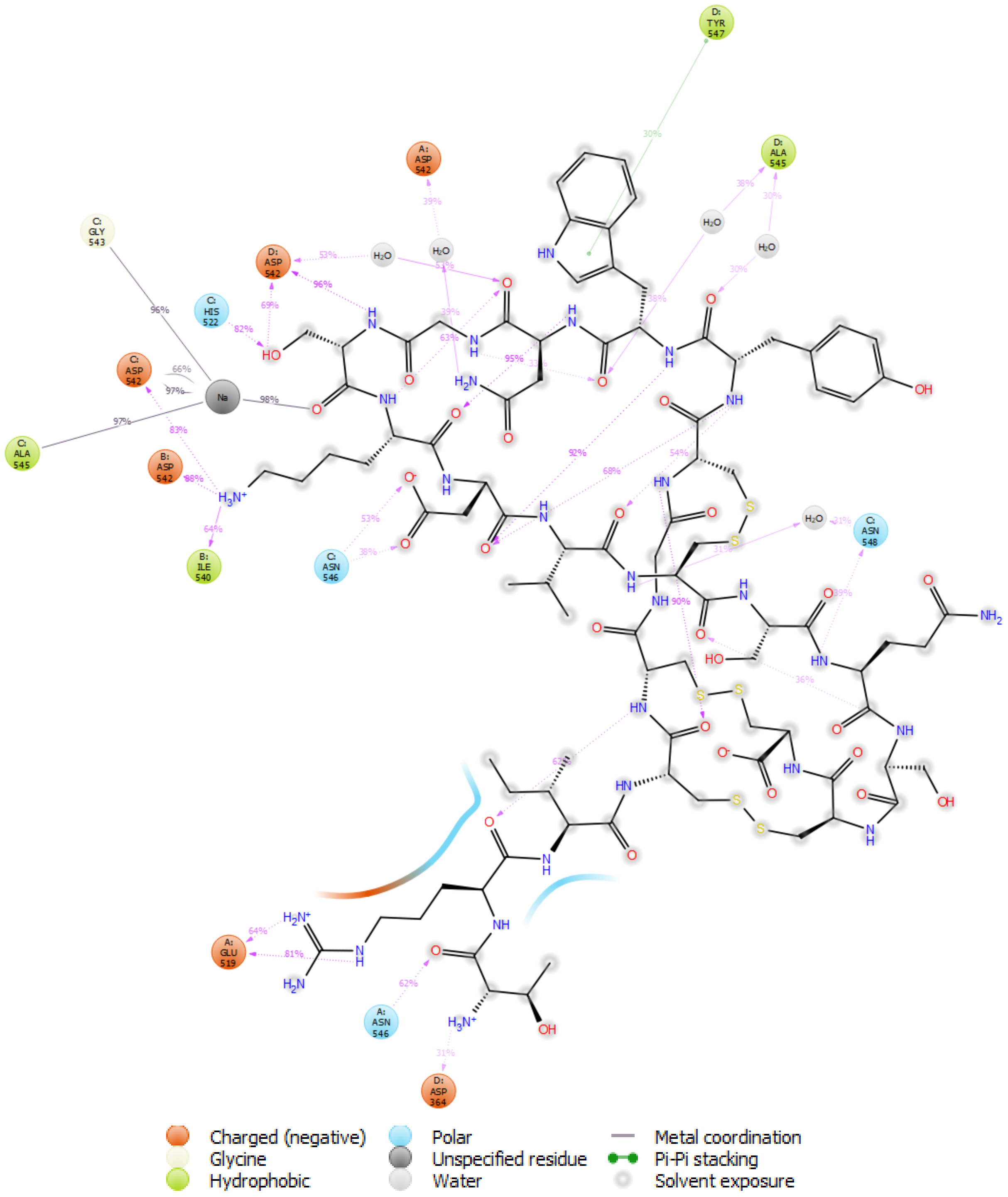

Figure S10. A schematic of detailed ligand Tv1 atom interactions with the hTRPV6 residues over 150ns molecular dynamics simulation. Interactions that occur more than 30.0% are shown.

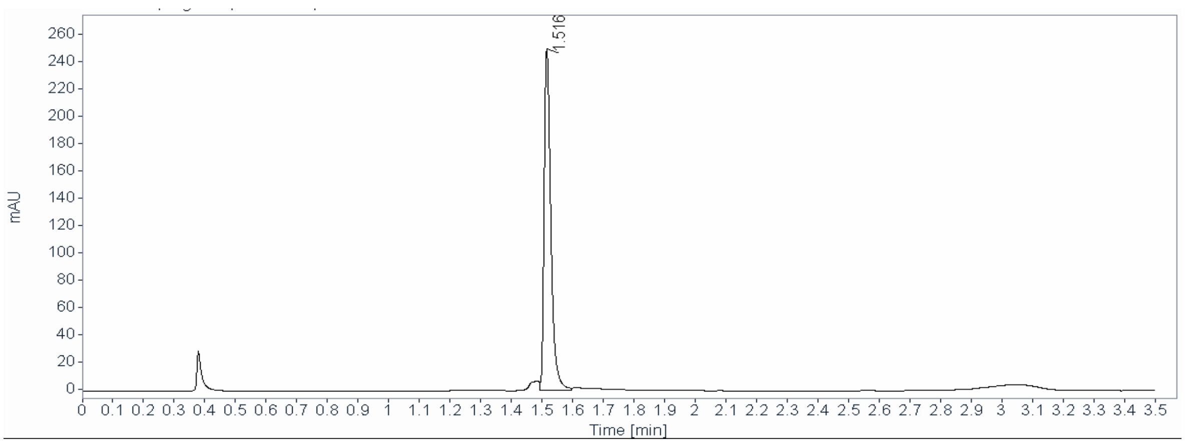

Figure S11a. RP-UHPLC chromatogram of Tv1 oxidized peptide at 214nm. The peak at retention time 1.51 min shows the presence of pure oxidized peptide.

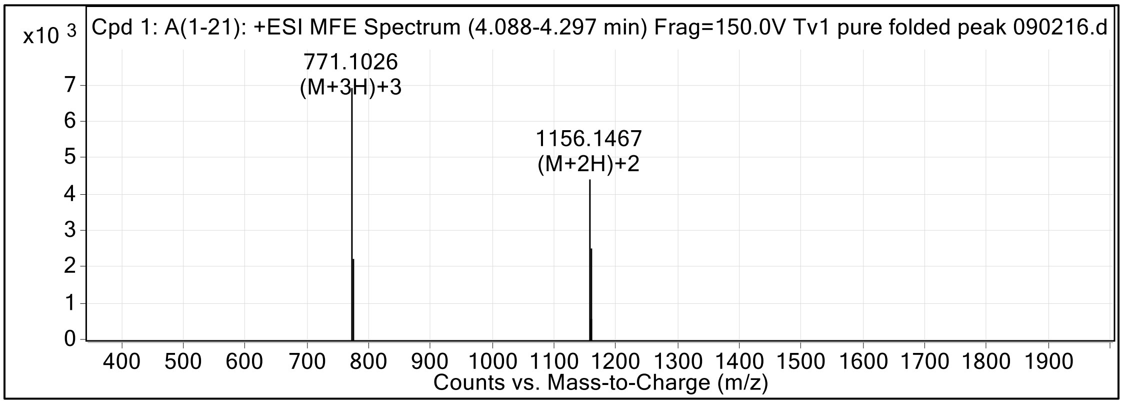

Figure S11b. Mass spectrum of oxidative folded Tv1 peptide. Expected molar mass of the peptide is 2308.825Da and the observed molar mass was found to be 2309.285Da.

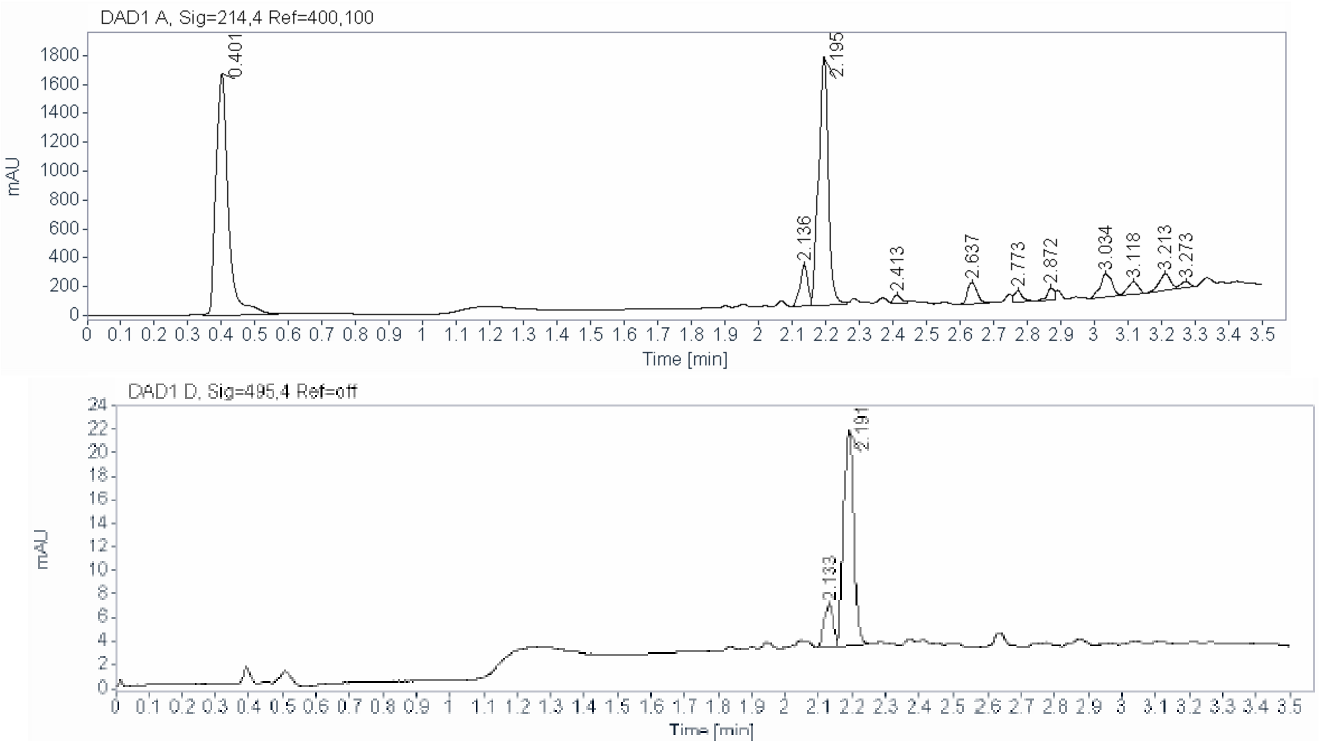

Figure S12a. RP-UHPLC of Tv1-Fam peptide. Signal at 214nm shows the peptide presence at 2.195 min retention time and at 495nm shows the labelling with the 5,6 carboxyfluorescein.

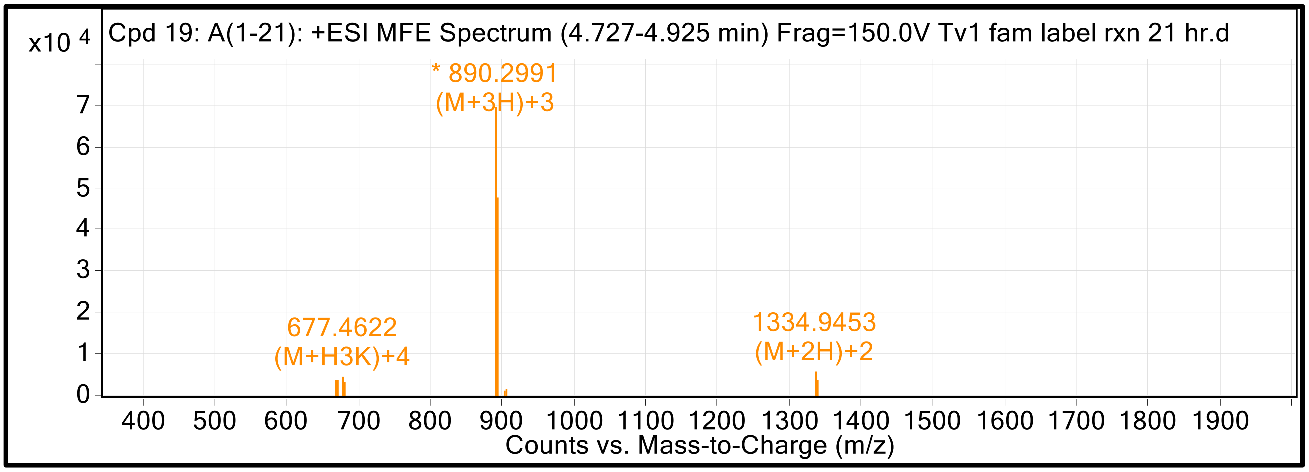

Figure S12b. Mass spectrum of oxidative folded Tv1-Fam peptide. Expected molar mass of the peptide is 2666.872Da and the observed molar mass was found to be 2666.870Da.

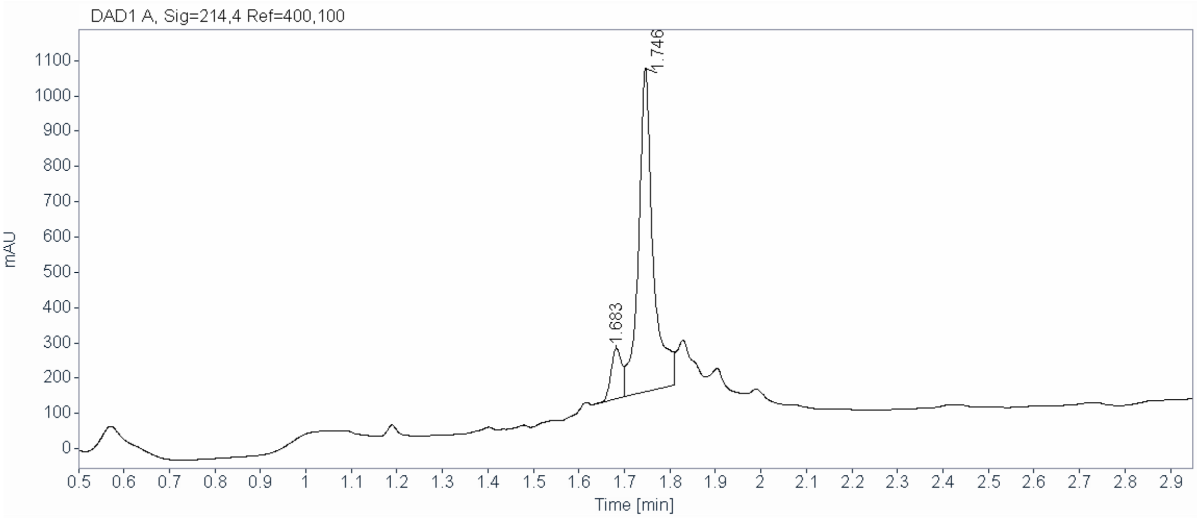

Figure S13a. RP-UHPLC chromatogram of truncated soricidin peptide (a TRPV6 inhibitor) at 214nm. The peak at retention time 1.746min shows the presence of peptide.

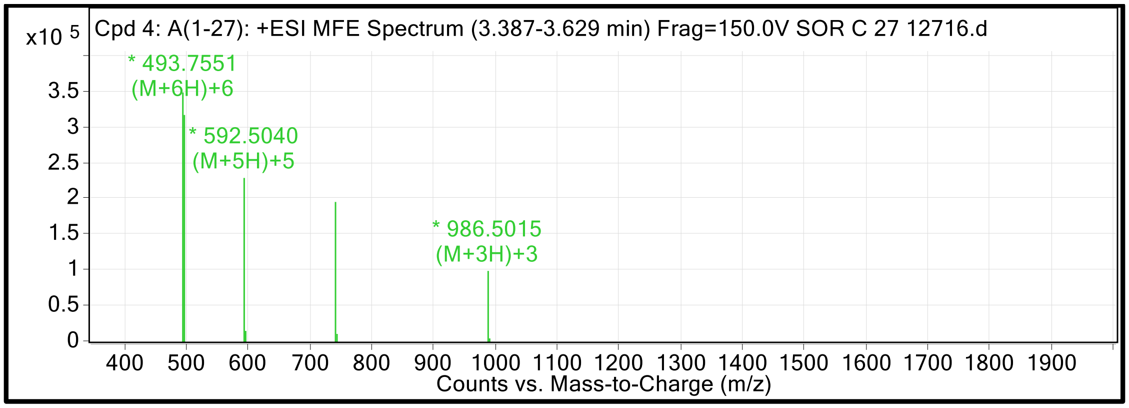

Figure S13b. Mass spectrum of chemically synthesized truncated soricidin peptide (a TRPV6 inhibitor) The expected molar mass of the 27residue peptide is 2955.476Da and analysis showed the observed molar mass of 2955.479Da

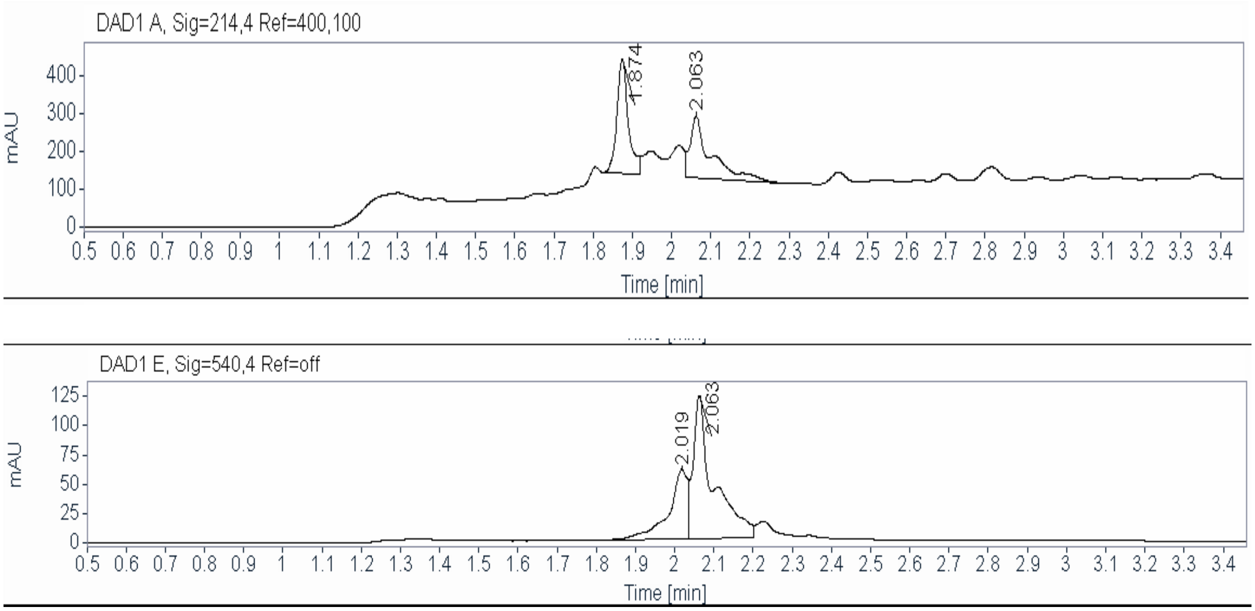

Figure S14a. RP-UHPLC of TrunSorC-Rho peptide. After conjugation reaction with Rhodamine-NHS ester, complete labelling was not obtained but sufficient labeling was done for IFA. At 214nm peak at retention time 1.87min shows the presence of unlabeled peptide and peak at 2.06min shows the presence of labeled peptide which was confirmed with the traces at 540nm.

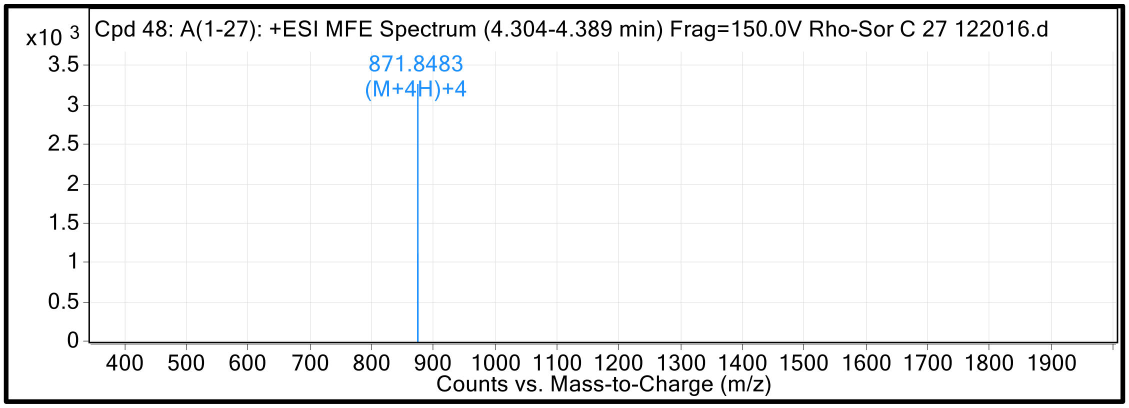

Figure S14b. LC-MS analysis of truncated soricidin-Rho peptide. m/z of 871.8483 is the peak for +4 molar mass 3481.460Da which matches with the expected mass 3481.645 Da of the peptide

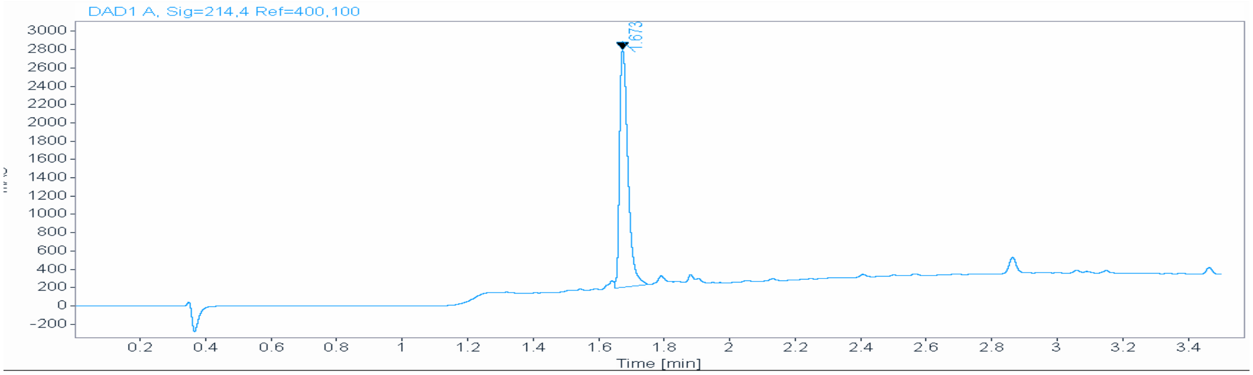

Figure S15a. RP-UHPLC of chemically synthesized ACP peptide. A negative control peptide was synthesized for IFAs. Peak at retention time 1.67min shows the presence of pure peptide.

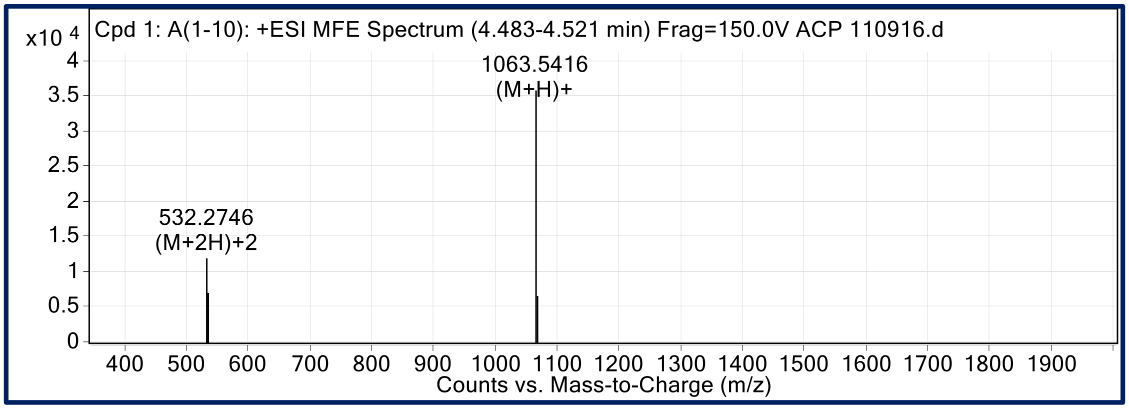

Figure S15b. LC-MS analysis of ACP peptide. The expected mass of the peptide is 1062.534Da and the observed mass was found to be (M+1) 1062.534Da.

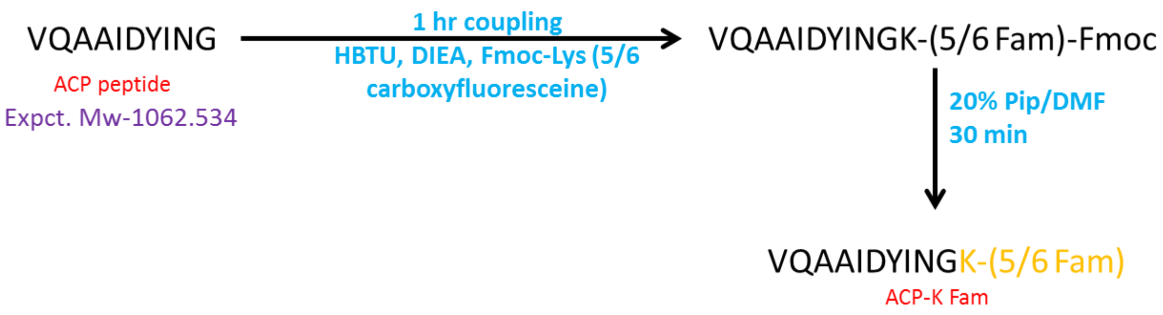

Figure S16a. Reaction scheme of synthesizing ACPK-Fam from ACP peptide.

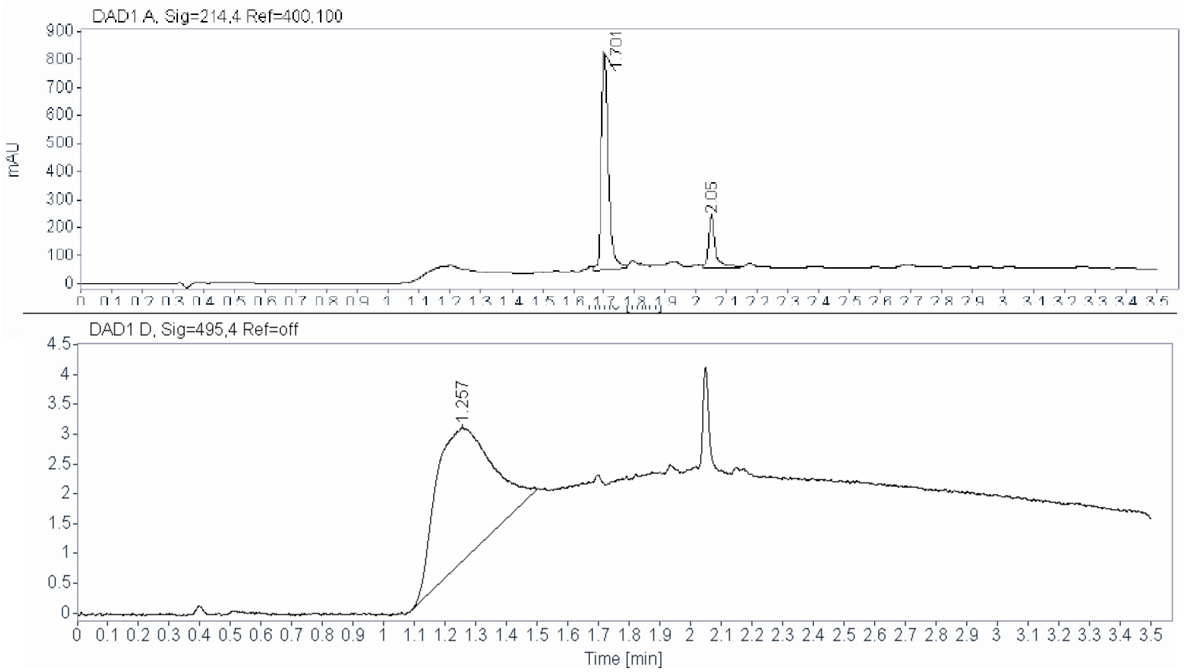

Figure S16b. RP-UHPLC of chemically synthesized ACP-K-Fam. Signal at 214nm at a retention time of 1.70 min shows the presence of ACP peptide and at 2.05 min shows the ACPK-Fam peptide which was confirmed by the signal at 495nm for Fam.

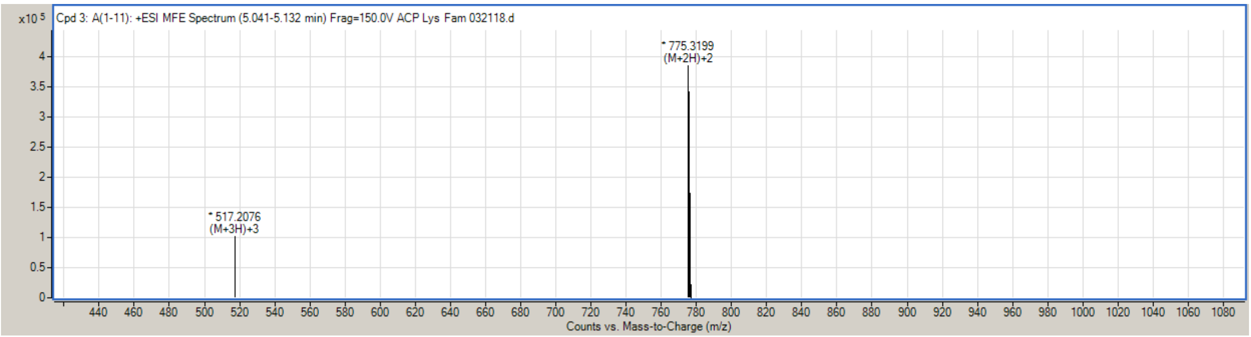

Figure S16c. LC-MS analysis of chemically synthesized ACPK-Fam peptide. HPLC peak of retention time 2.05 min showed the mass of 1548.62Da which is the expected mass of the ACPK-Fam.

Table S1. Statistical analysis of MMGBSA binding energy score during 150ns simulation

6B08/Tv1 (n=51)	6BO8/TruncSorC (n=51)
-84.3±9.5	-61.7±12.2
